# Therapeutic and Industrial Applications of Curdlan With Overview on Its Recent Patents

**DOI:** 10.3389/fnut.2021.646988

**Published:** 2021-06-28

**Authors:** Vinay Chaudhari, Harpal Singh Buttar, Siddhi Bagwe-Parab, Hardeep Singh Tuli, Amisha Vora, Ginpreet Kaur

**Affiliations:** ^1^Shobhaben Pratapbhai Patel School of Pharmacy and Technology Management, Shri Vile Parle Kelavani Mandal's Narsee Monjee Institute of Management Studies, Mumbai, India; ^2^Department of Pathology and Laboratory Medicine, Faculty of Medicine, University of Ottawa, Ottawa, ON, Canada; ^3^Department of Biotechnology, Maharishi Markandeshwar (Deemed to be University), Mullana, India

**Keywords:** curdlan sulfate, succinoglucan, immunomodulatory activity, anti-viral, anti-malaria, anti-cancer, functional food, β-(1,3)-glucan oligosaccharides

## Abstract

Curdlan is an exopolysaccharide, which is composed of glucose linked with β-(1,3)-glycosidic bond and is produced by bacteria, such as *Alcaligenes* spp., *Agrobacterium* spp., *Paenibacillus* spp., *Rhizobium* spp., *Saccharomyces cerevisiae, Candida* spp., and fungal sources like *Aureobasidium pullulan, Poria cocos*, etc. Curdlan has been utilized in the food and pharmaceutical industries for its prebiotic, viscosifying, and water-holding properties for decades. Recently, the usefulness of curdlan has been further explored by the pharmaceutical industry for its potential therapeutic applications. Curdlan has exhibited immunoregulatory and antitumor activity in preclinical settings. It was observed that curdlan can prevent the proliferation of malarial merozoites *in vivo*; therefore, it may be considered as a promising therapy for the treatment of end-stage malaria. In addition, curdlan has demonstrated potent antiviral effects against human immunodeficiency virus (HIV) and *Aedes aegypti* virus. It has been suggested that the virucidal properties of curdlans should be extended further for other deadly viruses, such as severe acute respiratory syndrome (SARS), Middle East respiratory syndrome (MERS), and the current severe acute respiratory syndrome coronavirus-2 (SARS-CoV-2/COVID-19). The prebiotic property of curdlan would confer beneficial effects on the host by promoting the growth of healthy microbiota in the gut and consequently help to reduce gastrointestinal disorders. Therefore, curdlan can be employed in the manufacture of prebiotics for the management of various gastrointestinal dysbiosis problems. Studies on the mechanism of action of curdlan-induced suppression in microbial and tumor cells at the cellular and molecular levels would not only enhance our understanding regarding the therapeutic effectiveness of curdlan but also help in the discovery of new drugs and dietary supplements. The primary focus of this review is to highlight the therapeutic interventions of curdlan as an anticancer, anti-malaria, antiviral, and antibacterial agent in humans. In addition, our review provides the latest information about the chemistry and biosynthesis of curdlan and its applications for making novel dairy products, functional foods, and nutraceuticals and also details about the recent patents of curdlan and its derivatives.

## Introduction

Exopolysaccharides are natural polymers of high-molecular-weight residual sugar polymers secreted by a wide variety of non-pathogenic microorganisms, including bacteria, yeast, and fungi. The extracellular natural polymeric substances belong to the carbohydrates category and provide many important health benefits, such as promotion of digestibility, biocompatibility, and endurance of long-term stability over a wide range of pH and environmental temperatures (1). Recently, researchers have found promising applications of natural polymeric substances, for example, curdlan, in the pharmaceuticals, food packaging, making biofilms, and in the dairy industry where they improve the viscosity and texture of the food products (2). Overall, curdlan-type exopolysaccharides have many emerging potential applications in making nutraceuticals, functional foods, cosmetics, drugs, and dairy products (3).

### Physical Properties of Curdlan

Curdlan is a water-insoluble exopolysaccharide secreted by a wide variety of non-pathogenic bacteria, yeast, and fungi as shown in **Table 2**. It is comprised of a homopolymer of D-glucose-linked with β-(1,3)-glucan (12). The unique thermal characteristics of curdlan are utilized in biofilm making to improve water barrier capacity and thermal stability in single- and multilayer food packaging (13). Apart from its high thermal stability, curdlan also possesses stability at lower freezing temperatures, and hence is used in frozen foods packaging. According to recent reports, curdlan biofilms with 5% caprolactam have been shown to possess better mechanical strength and other physical properties as compared to other films (14). Curdlan can form two types of gels based on their thermal properties, *viz*., low-set gel and high-set gel (15). The low-set curdlan gel is produced when the aqueous solution of curdlan is heated up to 60°C and then cooled to 40°C in a thermally reversible process. On the other hand, the high-set curdlan gel is generated when the aqueous solution of curdlan is heated >80°C in a thermally irreversible process (16). In chemical terms, there is a cross-linking between the curdlan micelles in the low-set gel, which are occupied by single-helix molecules through hydrogen bonds, whereas the curdlan micelles are cross-linked by a triple-stranded helix through hydrophobic interactions in the high-set gel (17). Curdlan gels are used for the encapsulation of a number of drugs like indomethacin, salbutamol sulfate, and prednisolone to provide sustained release of these drugs (18). They are also used for grafting copolymers in the pharmaceutical industry (19).

Curdlan is one of the emerging exopolysaccharide, which was discovered and studied by (14, 20). It has the ability to produce polysaccharide-based membrane film, which has proven effective in possessing gas barrier properties (20, 21). Due to its non-toxic attributes, curdlan was approved as a food additive by the US Food and Drug Administration (22). Prebiotics are non-digestible oligosaccharides that promote the growth of beneficial bacteria in the gut. Curdlan being an oligosaccharide has a prebiotic potential and therefore can be used to promote the growth of useful intestinal microbiota (23).

Curdlan has shown a wide range of pharmacotherapy functions, such as anti-HIV, immunomodulation, and antitumor activity (24). A recent study revealed that curdlan can inhibit the invasion of malarial merozoites and therefore can be considered as a possible auxiliary candidate for treating intense malaria (25). Additionally, curdlan has exhibited potential antiviral activity against human immunodeficiency virus (HIV) and Dengue fever virus (26). In view of its virucidal activities, curdlan may be considered a potential candidate against other deadly viruses, such as severe acute respiratory syndrome (SARS), Middle East respiratory syndrome (MERS), and the current severe acute respiratory syndrome coronavirus-2 (SARS-CoV-2/COVID-19).

There are many published reports regarding the therapeutic properties and excipient properties of curdlan (26), but the underlying mechanism of curdlan at the cellular and molecular level remains unknown. This review is intended to highlight the applications of curdlan in drugs and cosmetics, nutraceuticals, and functional foods and in potential industrial usages. In addition, our review provides the latest information regarding the chemistry and microbial sources used for the biosynthesis of curdlan, along with the recent patents applied by the food and drug manufacturers for the utility of curdlan.

## Chemistry of Curdlan

### Chemical Structures/Formulas of Curdlan Products

Chemical structures and formulas of curdlan compounds are shown in Table 1. The analytical studies have confirmed that curdlan is a D-glucose homopolymer linked in the manner of β-(1,3)-glycosidic bond (30). Curdlan is unbranched and has an average degree of polymerization of around 450. In an alkaline solution of 0.3 Mol, the molecular weight of curdlan is in the range of 5.3 × 10^4^ to 2.0 × 10^6^ Da. The bacterial and fungal species produced curdlan, the average weight lies between 2.06 × 10^4^ and 5.0 × 10^6^ Da (31).

**Table 1:** Structural forms and chemical formulas of curdlan produced by different bacterial species.

**Molecular form**	**Chemical formula**	**Source**	**References**
(1, 3)-β-D-glucans (linear glucan)	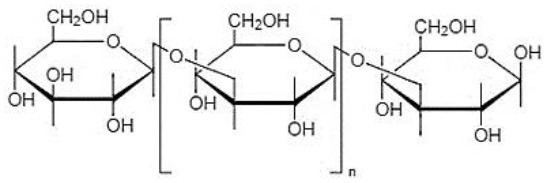	*Agrobacterium sp*.	(27)
(1, 3, 1, 2)-β-D-glucan (branched glucan)	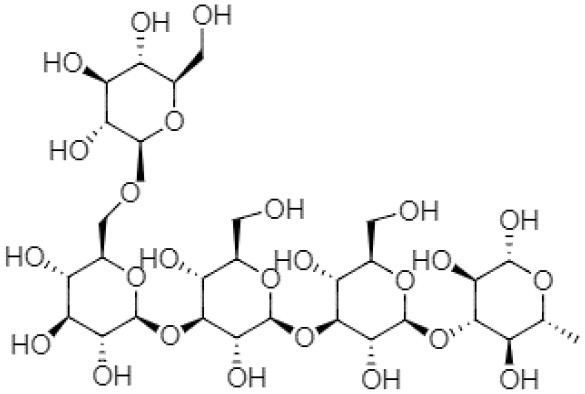	*Streptococcus sp*.	(28)
β-(1, 3, 1, 6)-β-D-glucan (cyclic glucan)	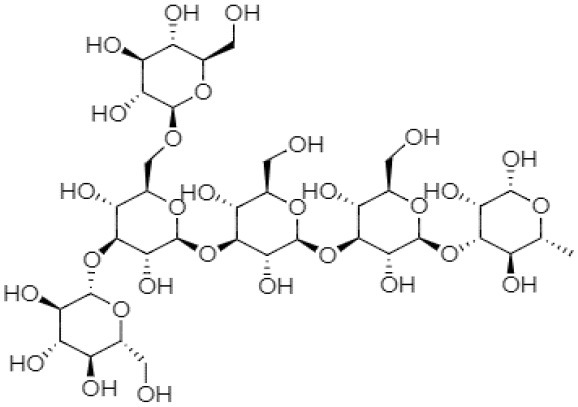	*Rhizobium sp*.	(29)

The general molecular formula of curdlan is (C_6_H_10_O_5_)_*n*_. It consists of approximately 135 residues of glucosyl, and the infrared spectrum shows a peak of β-arrangement gel at 890 cm^−1^, while α- arrangement gel shows a peak at 840 cm^−1^ (32–34).

There is a range of structural variations within the class of β-(1,3)-glucans polysaccharides (35). Curdlan falls into the category of neutral gel having β-(1 → 3)-glycosidic linkage or may also contain few inter- and intra-chain (1 → 6)-linkages, which contain almost 10% succinate known as succinoglycan (36). Curdlan is produced in three different structural forms by the bacterial species (28).

### Molecular Conformers of Curdlan

The molecular structures of curdlan in the aqueous systems have been determined by several investigators. For instance, Zhang et al. (37) identified three soluble curdlan conformers, including single-helix, triple-helix, and random coil. The single-stranded curdlan is composed of 2–25 glucose units that exhibit better water solubility with improved permeability. Different biological activities are associated with short disordered curdlan chains (38). Some curdlans have a helical (ordered) conformation at low NaOH concentrations (37). Ikeda and Shishido demonstrated using atomic force microscopy that as alkali concentration increases from 0.19 to 0.24 N, curdlan conformation changes from helix conformation to random coil structure (39). Cheung et al. reported that in 0.3 mol NaOH, the ultrasonic degradation was found to be much faster than in 0.1 mol NaOH, suggesting that curdlan is more sensitive to the degradation in the random-coil conformation than in the helix conformation (40). Saitô et al. confirmed that curdlan is fully soluble in an alkaline solution having a concentration >0.2 N NaOH and exists as a random coil, but the polymer adopts an “ordered state” after neutralization, which consists of a combination of single and triple helices (41). Zhang and Nishinari reported that at low concentrations, curdlan exhibits a relatively extended flexible chain structure in dimethyl sulfoxide, whereas chain entanglement occurs at a higher concentration (42).

## Biosynthesis of Curdlan

### Microbial Source

Curdlan can be produced as exopolysaccharides by different types of microbes, for example, bacteria, yeast, and fungi. Some major microbes are involved in the biosynthesis of curdlan, and the yields achieved are shown in Table 2.

**Table 2:** Microbial species used in the biosynthesis of curdlan and the yields achieved by different species.

**Source**	**Microbial species**	**Concentration achieved**	**References**
Bacterial source	*Alcaligenes spp*.	72 g/L	(4)
	*Agrobacterium spp*.	41.24 g/L	(5)
	*Paenibacillus spp*.	6.89 g/L	(6)
	*Rhizobium spp*.	31 g/L	(7)
Yeast source	*Saccharomyces cerevisiae*	5–90%	(8)
	*Candida albicans*	ND	(9)
Fungal source	*Aureobasidium pullulan*	33 g/L	(10)
	*Poria cocos*	90% dry weight	(11)

*ND, not described*.

*Alcaligenes faecalis* is a Gram-negative bacterium, which can be isolated from the soil and can yield maximum curdlan. The NaOH solution was substituted by feeding ammonia water at an initial stage to control pH at 7.0, and then the NaOH solution was added to control pH at 5.6 for the curdlan production after ammonia was consumed. As a result, 72 g/L of curdlan was produced at controlled nitrogen condition (4). *Agrobacterium radiobacter* is also a Gram-negative soil bacterium, which yields 41.24 g/L of curdlan after 96 h of fermentation (43). Recently, the biological activity as an immune stimulator of peripheral blood mononuclear cells to increase the interferon-γ secretion was shown by curdlan developed by the mutant strain of *Agrobacterium* sp. ATCC 31750 (ATCC, Manassas, VI, USA), having a molecular weight of 300,000 Da and consisting exclusively of the 1,3-glucose residue linkage (44). *Paenibacillus polymyxa* is a Gram-positive bacterium, which at 50°C and at pH 7 yields 6.89 g/L of curdlan (6). It is used as a drug delivery carrier for the sustained release of drugs and as a support matrix for the immobilization of enzymes (45). *Rhizobium radiobacter* is another Gram-negative bacterium, which can yield 31 g/L of curdlan after 72 h of fermentation (46). A recent study confirmed that curdlan obtained from *R. radiobacter* is highly thermostable with excellent water-holding capacity and can be used as an antioxidant and prebiotic agent. However, further studies are needed to check the biological potential of curdlan produced from *R. radiobacter* (47).

### Biosynthesis of Curdlan

The biosynthesis of extracellular polysaccharides can be divided into three steps. First, substrate uptake for the intracellular formation of polysaccharides and extrusion from the cells. Second, through active transport and group translocation involving substrate phosphorylation, a carbohydrate substratum reaches the cell. Finally, the substrate is guided either along catabolic pathways or those that lead to the synthesis of polysaccharides.

**Figure 1:**
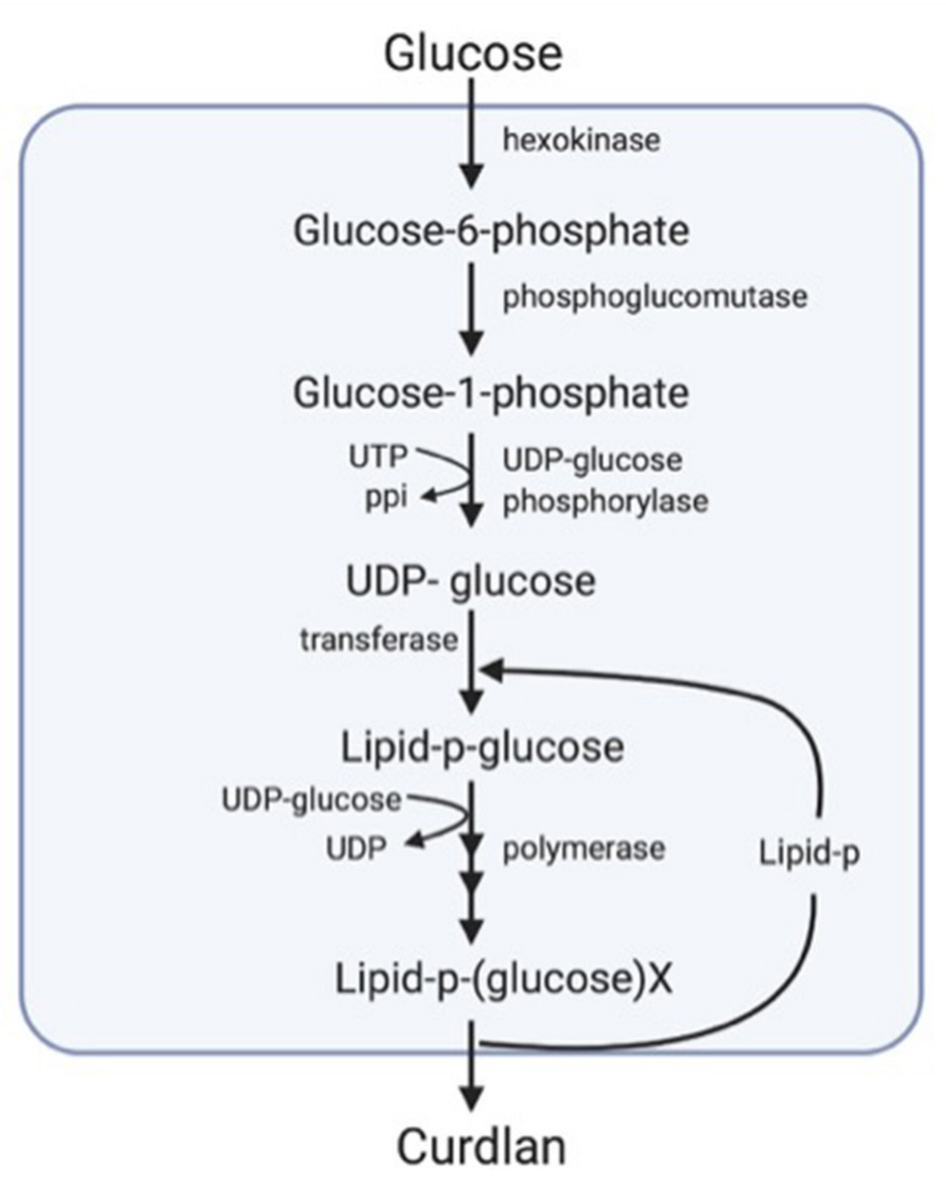
Diagrammatic representation of the biosynthesis of curdlan (33).

In the biosynthesis of curdlan, uridine diphosphate (UDP)-glucose constitutes the primary precursor that is synthesized *via* the conversion of glucose-1-phosphate to UDP-glucose by the enzyme UDP-glucose pyrophosphorylase. Later, the polymer structure occurs by the combination with the transition of monosaccharides to the carrier lipid from UDP-glucose. Subsequently, the polymer is extruded from the cells after further chain elongation (Figure 1) (48).

A study conducted by Mohsin et al. showed that curdlan can be synthesized from the orange peels using *A. faecalis*. The cocktail mixture included about 78% water, 10% soluble sugars, 35% cellulose, and 15% pectin from fresh orange peels. However, because of high moisture content, the cost for the preservation of curdlan is high, and hence drying process is an effective approach to store peels. By this technique, the dried orange peels yielded about 23.24 g/L of curdlan. The fermentation substrate showed significant effects on the development of curdlan and presented a novel engineered approach for the production of curdlan in an environment-friendly manner (49).

## Applications of Curdlan

### Food and Dairy Products

Curdlan gel is a colorless, odorless, and non-toxic substance that has water retention ability. All these versatile properties have led to its use as a food additive in the dairy industry (50, 51). Since curdlan is not easily hydrolyzed by the digestive enzymes, it can be added in prebiotics and to reduce calories and to promote healthy gut microbiota, and thus prevent obesity (52). It is also used as an additive in the preparation of frozen foods, pasta, and canned meat (53).

The hydrothermal hydrolysis of curdlan creates 1,3-gluco-oligosaccharides (GOS), which have proven beneficial in the production of functional foods and biopharmaceuticals (54). On account of the bacteriostatic, biocompatible, biodegradable, and hydrophilic characteristics, the quaternary ammonium salt solution of curdlan (Qcurd) is widely used along with its derivatives to encapsulate several bioactive food components (55). Several curdlan-induced benefits in food and dairy products are summarized in Table 3.

**Table 3:** Advantages of adding curdlan in foods and dairy products.

**Food type**	**Advantages of adding curdlan**	**References**
Noodles and pasta	1. Modified texture 2. Improved firmness 3. Improved shaping qualities	(56)
Meat and meat products	1. Heat and freeze-thaw stability 2. Maximized moisture retention at processing temperature 3. Improved functional properties of low-fat model sausages 4. Chilled meat preservation (curdlan film)	(57) (58)
Coating system and batter	1. Heat and freeze-thaw stability 2. Improved batter pickup and cooking loss	(59)
Milk and milk products	1. Increased viscosity of skimmed milk 2. Increased quality of milk 3. Retention of shape of ice-cream 4. Development of low fat whipped cream	(60) (61)

### Therapeutic Products

Biologically active β-(1,3)-glucan oligosaccharides were developed by the combination of heat treatment and hydrolysis of curdlan (62). *In vitro* batch fermentation using human fecal microorganisms showed that the bioactive oligosaccharides significantly increased the population of *Lactobacillus* in the gut and hence can be considered as a potential prebiotic (63). *In vivo* studies suggested that glucan oligosaccharide enhanced immunoregulatory activity in cyclophosphamide (CTX)-induced immunosuppressed mice by stimulating the release of nitric oxide (NO), cytokines, and increased immunoglobulins release and splenic lymphocyte proliferation (64).

Curdlan itself and GOS activate macrophages *via* complement receptor 3 (CR3) and toll-like receptor 2 (TLR2) by mitogen-activated protein kinase (MAPK) pathway and nuclear factor NF-κB pathway. The activated macrophages were found to be non-toxic or compatible with bone marrow-derived macrophages isolated from healthy male C57BL/6J mice. The GOS promoted M1 phenotype polarization of bone marrow-derived macrophages by upregulation of CD11C and CD86 expressions (65). In view of these findings, GOS may be considered safe to enhance immune responses when added to the development of functional foods (66).

Curdlan sulfate (CRDS) is a sulfated glycoconjugate that is biochemically similar to rosette inhibiting drugs like heparin and has been tried for treating severe malaria in 18 African children. CRDS inhibited erythrocyte rosette formation *in vitro* at 10–100μg/ml in *Plasmodium falciparum* laboratory strains and revealed anti-malarial activity in clinical isolates (67). Palm sulfopropyl (SP) and palm carboxymethyl/sulfopropyl (CM/SP) curdlan derivatives were able to potentiate the action of actinomycin D or doxorubicin in B16 tumor cells by inhibiting HEp-2 tumor cell growth and by upregulating doxorubicin and actinomycin D cytostatic activities (68). Further clinical trials are needed to establish the long-term safety and efficacy of CRDS in humans.

Barbosa-Lorenzi et al. found that curdlan acts as an agonist for the fungi receptor dectin-1 and induces selective mast cell degranulation without provoking synthesis of leukotriene and cytokine. Hence, curdlan may be employed against fungal infections in human skin (69). Curdlan pellicle produced by *Gluconacetobacter xylinus* is one of the best bio-based materials because of its super network structure and unique physiochemical properties and could be used for a wide range of clinical and tissue engineering applications (70).

#### Role of Curdlan as an Adjuvant

In order to enhance the immune response, adjuvants are added to vaccines to produce more antibodies and long-lasting immunity, thus minimizing the required antigen dose. In several studies, β-D-glucan has proven useful as an adjuvant carrier as it is a natural non-digestible polysaccharide that can be easily detected when bound to receptors, such as dectin-1. There is emerging interest in exploring the ability of curdlan to gain endogenous information in the desired organs or cells (71). Protective immunity to *Staphylococcus aureus* was observed by vaccination of mice with fungal β-glucan particles (GP) loaded with *S. aureus* antigens. The findings suggest that the glucan particles vaccine system has the potential as a novel approach for developing vaccines against *S. aureus* (72). Chemo- and radio-therapies cause several side effects in patients with cancer. Neutropenia is one of the most significant side effects of chemotherapy, which disrupts the process of blood formation in the bone marrow and can cause lymphocytopenia, thrombocytopenia, and granulocytopenia and consequently leading to immune system suppression (73). Curdlan represents a novel and promising therapeutic approach in treating cervical cancer and can be used as an adjuvant to reduce side effects of cancer chemotherapy treatment and help in recovering from compromised hematopoiesis in the therapy injured bone marrow (74).

In clinical trials, β-(1,3)-glucan has been used as adjuvant therapy with significant beneficial effects on the survival and quality of life of patients with cancer through the activation of the immune system (75). In comparison with the aluminum adjuvant, curdlan increased the immunogenicity of the hepatitis B vaccine and promoted an immune response in mice by increasing the specific antibody response to hepatitis B surface antigen (HBsAg) (76). Sulfated yeast β-glucan showed an excellent adjuvant effect on the influenza vaccine (H5N1) in a mouse model. The anti-influenza vaccine increased the influx of macrophages and dendritic cells, increased antigen-specific T cell levels, and induced splenocyte proliferation in mice (77). In another study done on mice, curdlan alone substantially boosted the immune response against influenza through stimulation of lymphocyte proliferation and enhanced cytokine production (78). Well-designed clinical studies are warranted to verify the results obtained in animal models.

#### Curdlan as an Antioxidant and Anti-inflammation Agent

It has been hypothesized that β-(1,3)-glucan products are capable of increasing the lifespan, slowing the emergence of age-related biomarkers and exerting the antioxidant impact of *Nothobranchius guentheri* and hence may be potentially useful for promoting health and well-being and extension of lifespan in humans (79). Recent studies have shown that beta-glucans can decrease hyperglycemia, hyperlipidemia, and high blood pressure. By minimizing oxidative stress-induced mitochondrial damage, curdlan/β-(1,3)-glucan may contribute to new approaches to manage diabetes mellitus associated complications, neurodegenerative disorders, and cardiovascular diseases (80). It has also been suggested that curdlan obtained from *S. cerevisiae* protects the human plasma components from strong biological oxidants and inflammatory mediators due to its free radical scavenging capability (81). A study conducted on isolated rat hepatocytes showed that curdlan obtained from the fungal source can capture free radicals and may be a possible candidate for prophylaxis and treatment against depleted uranium toxic effects (82). Furthermore, the systemic administration and local application of beta-glucan were effective against burn-induced oxidative tissue damage in rats (83).

#### Protection Against Hyperglycemia in Patients With Diabetes Infected With COVID-19

Curdlan obtained from yeast *Aureobasidium pullulans*, also known as biological response modifier glucan (BRMG), has the ability to bring the blood sugar levels under control and has the potential in enhancing and regulating immune parameters. Fasting plasma glucose (FPG) level is one of the indicators of COVID-19 infection, which causes high mortality among patients with diabetes and is responsible for causing serious pathological complications in vital organs. Consequently, BRMG may help to control FPG levels and may be used as a preventive measure against the COVID-19 virus (84). COVID-19 has also been linked to an increased risk of mortality in patients with comorbidities including diabetes. Diabetics have a higher risk of disease severity in COVID-19 because they have a higher incidence of inflammatory processes due to persistent glucose recognition by C-type lectin receptors (84). Reduced PI3K/Akt activity is a crucial factor in the development of diabetes. Dectin-1 (belongs to the C-type lectin receptors family), complement receptor-3, lactosylceramide, and scavenger and toll-like receptors are among the receptors that BRMGs activate. The immune system of patients with diabetes is typically impaired. The immune system can be boosted by BRMG (85). Diabetes is linked to glycation-induced chronic inflammation of beta cells (β-cells) in the pancreas and elevated hemoglobin A1C (HbA1C) and glycated hemoglobin levels with clinical relevance as a marker of diabetes diagnosis and glycemic regulation. The immune-boosting activity of BRMG is linked to its potential anti-glycation effect (normalization of HbA1c) in patients with diabetes (86). In addition, the development of a hypercoagulable prothrombotic state is favored by an imbalance between coagulation and fibrinolysis, with increased levels of clotting factors and relative inhibition of the fibrinolytic system, endothelial dysfunction, and enhanced platelet aggregation and activation, all of which are complications of diabetes, explains the high risk of patients with diabetes to COVID-19 in terms of intensity/severity (85). Yeast BRMGs have been found to regulate the coagulation activation by controlling the cytokine activity and reversing diabetes-induced oxidative stress, which causes clotting parameters to be disrupted due to increased platelet aggregation and thrombin levels (87). Under the prevailing circumstances, it is of utmost importance to reduce COVID-19-induced mortality and serious complications such as coagulopathy, especially among the vulnerable populations (85). In order to fight against COVID-19-like pathogens, BRMG may be used to boost the immune system in humans. BRMG may also prevent blood clotting from occurring due to radiation exposure or diabetes-induced oxidative stress that enhances platelet aggregation and increases thrombin levels. It was reported that BRMG increases the level of IL8, while decreases the levels of several interleukins (IL-1β, IL-2, IL-4, IL-6, and IL-12), tumor necrosis factor (TNF)-alpha, interferon-gamma (IFN-γ), and active soluble fas-ligand (sFasL) levels, thereby maintaining the effective optimal defense against hyper-inflammation-free viral infections (87). Hyperinflammation results by preventing the onset of apoptosis and by increasing the development of sFAS and by suppressing the cytokine storms through a cascade of molecular events involved in activating Treg cells and reducing IL6, and at the same time preventing the chemoattraction of monocytes and macrophages, T cells, NK cells, and dendritic cells by reducing CXCL10 and CCL2 cells (88). Overall, BRMG enhances the immune function in the upper respiratory tract to protect against microbial pathogens (89). The immune boosting activity is one of the most important attributes of β-glucans. Keeping in view these observations, further clinical trials are required to validate the antiviral and immune system prophylaxis of BRMG.

The virucidal properties of BRMG should be explored further for other deadly viruses, including the lethal viruses like SARS, MERS, and the virulent strains of SARS-CoV-2/COVID-19. Although SARS-CoV-2 is primarily a respiratory virus, the viral host receptor angiotensin-converting enzyme 2 (ACE2) is found in the cytoplasm of gastrointestinal epithelial cells, and the viral nucleocapsid protein is found in the cytoplasm of rectal, duodenal, and gastric epithelial cells, suggesting that the intestine may play a role in COVID-19 pathogenesis and may be a possible route of infection. β-glucans with immune effects in the intestine could thus be a beneficial supplementation method for the COVID-19 treatment (90). A β-glucan extract from the edible shiitake mushroom *Lentinus edodes* was recently confirmed to have a differential *in vitro* immunomodulatory and pulmonary cytoprotective effects, suggesting that it may be used to treat COVID-19. According to the findings, β-glucans delivered as a customized cocktail may be a good match for a potential nutraceutical-based COVID-19 intervention (91). In a randomized, placebo-controlled, and double-blind trial, Mah et al. found that the severity of upper respiratory tract infection (URTI) was lower among marathon runners (*n* = 76) who consumed dairy beverages containing 250 mg soluble baker's yeast beta-glucan (Wellmune® Austin, TX, USA) as opposed to athletes given placebo (*n* = 133). The severity was evaluated by the Jackson index, and results showed that the yeast beta-glucan significantly reduced the severity of nasal discharge and URTI in marathon runners (92).

Therefore, the above evidence suggests the application of curdlan in the treatment of hyperglycemia in patients infected with COVID-19.

#### Wound Healing Properties of Curdlan

β-Glucans increase wound repair by increasing the infiltration of macrophages, which promote tissue granulation, collagen deposition, and skin re-epithelialization. Curdlan-based wound dressings have a great stability and resistance to wound protease, prevent bacterial infections, and are effective in wound healing. According to a clinical study conducted by Majtan and Jesenak, the application of 3% cream containing curdlan isolated from *S. cerevisiae* caused 55.23% reduction in ulcers after 90 days of treatment (93). In humans, fungal β-D-glucans reduce blood cholesterol and improve absorption of glucose in cells and thereby help in wound healing (94).

#### Immunomodulation Functions of Curdlan

Besides the above-mentioned usages, curdlan appears to have some other immunomodulation applications. Recently, Song et al. reported improvement in the *in vivo* humoral and cell-mediated immune responses in aged mice after the antigen challenge thereby suggesting the antiaging efficacy of curdlan (95). Curdlan derivatives also have anti-allergic activities, which have been verified in animal models and clinical trials in humans (96). Burg et al. observed that the daily oral administration of fungal β-glucan (400 μg/day) significantly reduced the influx of eosinophils into the lungs of ovalbumin challenged mice compared with control counterparts, thereby suggesting the protective action of curdlan against the development of asthma (97). The overactive type II helper T-lymphocytes response is thought to occur in individuals who experience allergic asthma attacks. It has been observed that β-(1,3)-glucan attenuates Th2 cytokines response by modulating the activity of macrophages, which produce pro-inflammatory mediators, such as prostaglandin E2, tumor growth factor, and IL-10 (98). Some investigators suggest that curdlan may be helpful in the prevention of chronic fatigue syndrome because it increases the proliferation of splenic lymphocytes and enhances the production of cytokines together with the stimulation of humoral and cellular immunity (99).

## Recent Patents of Curdlan

Besides other functional and biological properties, curdlan has been identified as an antiviral and antibacterial substance. As an immunomodulatory agent, curdlan can bind as a phagocytic receptor ligand and can act as dectin-1 agonist (69). In addition, curdlan can act as C-type lectin receptor and hence can achieve targeted immunotherapy (100). Apart from this, curdlan has unique gelling properties and thus can be applied to increase elasticity and the overall gel strength (13). Curdlan can also be used to form edible preservative films (14, 15). Recent patents of curdlan are mostly concentrated toward its immunomodulatory functions and improvement of physical properties of foods and drugs in the packaging processes. A comprehensive list of recent patents on curdlan, its applications, inventors, and patent holders are summarized in Table 4.

**Table 4:** Recent patents on curdlan showing patent holders, therapeutic products and a wide array of industrial applications.

**Sr. No**.	**Application year**	**Patent number**	**Title**	**Application of curdlan**	**Inventors**	**References**
1	2020-10-13	CN111758772A	Pomegranate fresh-keeping method	To form edible preservative film	Pang Fahu, Chang Wei	(101)
2	2020-10-09	CN110872360A	Preparation method of oxidized curdlan	To make oxidized polysaccharides	Chen Meiling, Ji Tianchen	(102)
3	2020-10-08	WO2020206354A1	Methods of depleting disease-causing agents via antibody targeted phagocytosis	Phagocytosis receptor ligand	Panagiotis FOTAKIS, Chanty Mariategue CHAN, Ruo Shi SHI, Adam Lewis SALLES, Nenad Tomasevic	(103)
4	2020-09-24	WO2020189840A1	Immunity enhancing adjuvant that can be administered in combination with oil emulsion, and vaccine composition for foot-and-mouth disease comprising same	Dectin-1 agonist	Jonghyun Park, Sumi Kim, Hyundong Cho, Byeonghan Kim	(104)
5	2020-09-24	US20200299502A1	Rubber-containing graft polymer, resin composition containing rubber-containing graft polymer, and shaped article of same	Grafting polymer	Misaki HAGI, Shinji Matsuoka, Masashi IIMORI, Tatsuhiro Hiraoka, Yuuta MAENAKA	(105)
6	2020-09-11	CN111646505A	Method for preparing nano-zirconia by using curdlan gel	Reduces the introduction of impurity ions	Jiang Bo, Luo Bing, Zhou Zhiyong	(106)
7	2020-09-01	CN111607367A	Temperature-sensitive gel plugging agent	Increases gel strength at high temperature with bentonite	Zhang Kun, Wang Min, Wang Leilei, Su Jun, Xu Shaoying, Zhu Li, Liu Xin, Ma Hong, Liu Qiang, Wang Hui, He Peng	(107)
8	2020-08-28	CN111588006A	Mushroom seafood slips and preparation method thereof	Hydrophilic colloid	Gao Xiangdeng	(108)
9	2020-08-18	CN111543626A	High-elasticity gel ball prepared by compounding curdlan and sodium alginate and preparation method thereof	Increases elasticity of gel ball with sodium alginate	Wang Jinyuan, Ai Zheng, Jin Zhu, Xu Geng	(109)
10	2020-08-07	CN111500366A	Preparation method and system of aroma-carrying gel	Aroma carrying gel	Li Chao, Wu Yiqin, Li Exian, Wang Qinghua, Li Xiangli, Su Yang, Hu Yan, Tang Jie, Cai Haocheng, Fan Duoqing, Xiong Wen	(110)
11	2020-08-06	US20200246478A1	Compounds for targeted immunotherapy	C-type lectin receptor	Lixin Li	(111)
12	2020-07-28	CN111448925A	Method for preventing and treating root knot nematode disease of fruit and vegetable vegetables	Base fertilizer	Qin Lijin, Zhang Zengming, Qian Cheng, Liu Lihong	(112)
13	2020-07-28	CN111454584A	Non-toxic environment-friendly degradable packaging material and preparation method thereof	Nontoxic, environment friendly and degradable packaging material film former	Liu Keyong	(113)
14	2020-07-03	CN111357885A	Complete feed and premix for promoting body shape and skeleton development of laying chicks and preparation method thereof	Rapid development of an immune system	Fu Xiuling, Zhu Wentao, Kong Xiangbin, Liu Hu, Niu Ziqing, Li Yan, Li Xiangjun, Ding Haifeng	(114)
15	2020-06-19	CN111296740A	Method for preparing dry rice noodles by using curdlan	Used as gelatinizer	Li Caiming, You Yuxian, Li Zhaofeng, Gu Zhengbiao, Chen Di, Ban Xiaofeng, Hong Yan, Cheng Li	(115)
16	2020-05-26	KR20200057625A	Grouting material including biopolymer-containing hydrogel to decrease negative skin fricton	Injection material for reducing secondary friction	Gyechun Jo, Youngman Kwon, Ilhan Jang, Lee Minhyung	(116)
17	2020-05-14	KR20200048509A	Composition for sublingual administration of curdlan inhibiting antigen specific immune responses	Inhibiting antigen-specific immune response	Jang Sun-young, Lee Eun-je	(117)
18	2020-04-28	CN111067085A	Compound polysaccharide cream and preparation method thereof	Polysaccharide paste preparation with good water holding performance	Zhang Xiaojian,Bu Guojian, Liu Junchang, Qian Yuan	(118)
19	2020-02-21	CN110810698A	Polyphenol oxidase inhibitor and application thereof	Polyphenol oxidase inhibitor	Chen Meiling, Ji Tianchen	(119)
20	2020-02-13	US20200048310A1	Compounds that bind to human immunodeficiency virus rev response element	Used in combination with HIV treatment agents	Yun-Xing Wang, Ping Yu	(120)
21	2020-02-04	CN110742232A	Preparation process of frozen cooked noodles	Modifier of the frozen cooked noodles	Liang Ying, Qu Zhuoting, Wang Jinshui, Zhu Mengfei, Liu Zhilei, Li Jie	(121)
22	2020-01-23	WO2020017456A1	Agent and method for improving physical properties of cheese-containing food	Physical property improver	Yusuke Matsuyama	(122)
23	2020-01-03	CN110638059A	Method for producing second-generation dietary fiber powder by liquid-phase wall-breaking grinding of soybean dregs	Improve the exchange capacity of dietary fibers and cations and functionality of products	Chen Yuewei, Chen Yu, Liu Hongfei	(123)
24	2019-10-08	CN110305232A	A kind of water-absorbent material and preparation method thereof based on modified gel polysaccharide	Water absorbent material	Han Jingfen, Sun Yingjian, Wei Shuai, Li Qingfeng, Pan Yiwen	(124)
25	2019-08-30	CN110183546A	A kind of curdlan water solubility antibiotic derivative and preparation method thereof	Modified curdlan antibiotic derivative	Han Jingfen, Zhao Ruichun, Gu Feng, Lu Zhimin	(125)
26	2019-07-23	CN110041537A	A kind of ionomer carboxymethyl curdlan gum physical hydrogel and preparation method thereof	As a polysaccharide hydrogel improving cross-linking	Zhang Hongbin, Cai Zhixiang, Wu Min, Li Ruiqi, Wang Pengguang	(126)
27	2019-05-10	CN109730273A	It is a kind of using konjaku flour and curdlan as the preparation method of the blocky fat analog of matrix	Raw material for preparation of blocky fat analog of matrix	Liu Qian, Kong Baohua, Jia Xiwen, Cao Chuanai, Zhao Shenyi	(127)
28	2018-12-11	CN108976485A	A kind of curdlan and the compound flexible light-emitting film and preparation method thereof of rare earth	Improved luminescent properties and permeability (translucency)	Han Jingfen, Li Qingfeng, Zhao Ruichun, Sun Yingjian, Lu Zhimin	(128)
29	2018-07-31	PL415936A1	Bone scaffold based on−1,3-glucan (curdlan) and bioceramics and method for producing it	Bone scaffold	Katarzyna Klimek, Agata Przekora, Grazyna Ginalska	(129)
30	2018-03-06	CN107751973A	Curdlan improves the correlation technique of solubility property	Improving solubility property and utilization rate	Teng Yarong, Liu Yi	(130)
31	2018-01-26	CN107625961A	The O n-trimethyl chitosan chlorides nanoparticle of sulphation Curdlan polysaccharide/6 and its application in mucosal vaccine	As an immunoadjuvant	Wang Fengshan, Zhang Shu, Lu Lu, Li Pingli, Cheng Yanna	(131)
32	2017-10-19	WO2017178675A1	Cardboard container for active packaging of fresh fruit and vegetables and production method of same	Provide active antimicrobial coating with essential oils	Antonio Lopez Gomez	(132)
33	2017-05-17	CN106666551A	Method for modifying potato powder by using TG (Transglutaminase) compounded with curdlan	As stabilizer	Jiang Jianhua	(133)
34	2017-05-10	CN106616594A	Curdlan-containing food for preventing cold hernia abdominal pain and preparation method of curdlan-containing food	Prevents cold hernia and stomachache	Ling Aiping	(134)
35	2017-04-26	CN106578117A	Making method of chrysanthemum flower bean curd adopting curdlan as coagulator	As coagulator	Gao Xinhua	(135)
36	2017-02-02	WO2017017953A1	Food product for individuals with difficulty in chewing which is refrigeration and freezing tolerant and which can be cooked	Increases heat resistance	Toshiro Kobayashi, Keisuke Kinpira, Yoshimi Usami	(136)

## Conclusions And Future Directions

A wide variety of exopolysaccharides or curdlans are produced by using bacteria, fungi, and yeast. The unique physicochemical properties of soluble and insoluble curdlans have been found highly valuable materials in different industries, such as dairy, food manufacturing, cosmetics, and pharmaceuticals. As shown in Table 4, curdlan hydrocolloids play very important roles in making novel functional foods, pharmaceuticals, and dairy products like prebiotics. Some curdlans are dectin-1 receptor agonists and hence can be employed as adjuvant carriers for vaccines, and their immunomodulatory activity can be beneficial in cancer chemotherapy and prevention of autoimmune disorders. A number of curdlans have shown antiaging, antidiabetic, antioxidant, anti-inflammation, wound healing, and cardio- and neuro-protection capabilities. Some curdlans have revealed the potential for treating malaria, pathogenic bacteria, and viral diseases (HIV and SARS-CoV-2/COVID-19). However, further well-designed studies are needed to verify the preliminary results obtained in a limited number of clinical observations.

It is also important to evaluate the immunomodulatory activities of different curdlans in suitable animal models and randomized and well-controlled clinical studies in humans. More research is also warranted to understand the mechanism of action of curdlans at the cellular and molecular levels to develop novel products. In addition, there is a need to study the formulation characteristic of curdlans under different environmental conditions for creating stable formulations with long-lasting shelf lives.

The current patents on curdlan are mostly concentrated toward biopolymers, curdlan gels, polysaccharide-based membrane film, immunomodulatory functions, and improvement of physical properties of foods and drugs in the packaging processes. Microencapsulation and nanotechnology studies are needed for the development and delivery of antiviral, anticancer, and parenteral therapies of curdlan. Good manufacturing procedures and quality control methods must be used in making curdlan preparations.

## Author Contributions

GK, HB, and HT visualized the presented idea, contributed to manuscript writing, and supervised the project. VC, AV, and SB-P contributed in doing literature searches and prepared manuscript draft. GK and HB revised and approved the manuscript. All authors contributed to the article and approved the submitted version.

## Conflict of Interest

The authors declare that the research was conducted in the absence of any commercial or financial relationships that could be construed as a potential conflict of interest.
